# A Biomechanical Comparison Study of Plate–Nail and Dual-Plate Fixation in AO/OTA 41-C2 Tibial Plateau Fractures

**DOI:** 10.3390/bioengineering11080839

**Published:** 2024-08-17

**Authors:** Wei Xie, Deqing Luo, Li Xie, Lingqi Zhu, Liang Zhou, Kejian Lian, Dasheng Lin, Hui Liu

**Affiliations:** 1 Department of Orthopaedic Surgery, The Affiliated Southeast Hospital of Xiamen University, Zhangzhou 363000, China; xiewei12353@stu.xmu.edu.cn (W.X.); deqingluo2012@163.com (D.L.); xieli1995@stu.xmu.edu.cn (L.X.); lqzhu@stu.xmu.edu.cn (L.Z.); byronlioncocoa@163.com (L.Z.); gkxiaohe@163.com (K.L.); 2 Department of Orthopedic Surgery, Fujian Medical University Union Hospital, Fuzhou 350000, China

**Keywords:** tibial plateau fracture, internal fixation, intramedullary nail, plate, biomechanics

## Abstract

Background Context: This study’s purpose was to evaluate the biomechanical performance of plate–nail and dual-plate fixation for the treatment of AO/OTA 41-C2 tibial plateau fractures. Methods: Twenty synthetic tibias were selected and randomly divided into a plate–nail group (*n* = 10) and a dual-plate group (*n* = 10). After the artificial tibias were osteotomized to simulate AO/OTA 41-C2 tibial plateau fractures in both groups, the plate–nail and the dual-plate methods, respectively, were used for fixation, and then axial compression loading, three-point bending, torsion, and axial failure tests were carried out. The data of each group were recorded and statistically analyzed. Results: In the axial compression test, the average stiffness of the plate–nail group was higher than that of the dual-plate group (*p* < 0.05). The displacement generated in the plate–nail group was significantly smaller than that in the dual-plate group (*p* < 0.05). In the resisting varus test, the stress of the plate–nail group was significantly higher than that of the dual-plate group (*p* < 0.05). In the resisting valgus test, the stress of the plate–nail group was slightly higher than that of the dual-plate group, but the difference was not statistically significant (*p* > 0.05). In the static torsion test, the load applied to the plate–nail group was smaller than that of the dual-plate group when rotated to 5° (*p* < 0.05). In the axial compression failure test, the average ultimate load of the plate–nail group was significantly higher than that of the dual-plate group (*p* < 0.05). Conclusion: The treatment of AO/OTA 41-C2 tibial plateau fractures with plate–nail fixation is superior to that with dual-plate fixation in resisting axial stress and preventing tibial varus deformity, while dual-plate fixation has better resisting torsional ability.

## 1. Introduction

AO/OTA 41-C2 tibial plateau fractures are usually caused by high-energy injury, often accompanied by varying levels of soft tissue injury [1,2,3]. They are comparatively difficult to treat clinically, especially with that injury [4,5]. This difficulty is reflected in the conflict between a firm fracture fixation and soft tissue injury [6]. If internal fixation is not firmly established, the reduction in the fracture may be compromised, leading to the displacement of the articular surface. Consequently, the tibial plateau may not align properly with the femoral condyle, altering joint stress distribution during weight-bearing activities of the knee joint. This misalignment can result in secondary wear of the tibial plateau’s articular surface, ultimately progressing to articular cartilage degeneration. Such outcomes can severely impact the patient’s quality of life and work capacity. For firm fixation, its conventional treatment thereof is often performed with medial and lateral dual-plate fixation [7,8,9]. However, double incisions are more traumatic to soft tissues, and the rates of infection and flap necrosis with this method are increased significantly [10,11]. Additionally, the biomechanical strength of unilateral plate fixation or intramedullary nail fixation is insufficient, and clinical outcomes have been unsatisfactory [12,13]. If external fixation combined with limited internal fixation is taken, or if surgery is performed after soft tissue conditions are improved, the knee joint fixation time is longer, which can easily lead to complications such as knee stiffness and muscle atrophy. Moreover, although the medial and lateral dual-plate fixation of AO/OTA 41-C2 tibial plateau fractures provide adequate stability to allow an early range of motion (ROM), immediate full-weight bearing is not recommended [14]. Based on the above, there are many deficiencies in the usual treatment programs, resulting in the long treatment cycle of patients with such fractures, poor efficacy, and significant economic burden. Therefore, it is of great significance to provide a treatment method that can shorten the treatment time, provide early functional exercise and obtain a good fracture healing effect for clinicians and patients.

In a recent study, treatment with intramedullary nail fixation combined with unilateral plate fixation for AO/OTA 41-C2 tibial plateau fractures with poor soft tissue conditions on one side achieved good clinical results [15]. This technique uses an intramedullary nail instead of an internal buttress plate on the medial side to maintain tibial alignment and avoid tibial varus while ensuring the fixation strength of the proximal tibial fracture and avoiding the medial soft tissues [16,17]. However, to date, there have been no biomechanical studies to confirm whether this procedure can provide similar fixation effects to the treatment of AO/OTA 41-C2 tibial plateau fractures with medial and lateral dual-plate fixation. Therefore, we conducted biomechanical testing using artificial bones to compare the biomechanical efficacy of plate–nail versus dual-plate fixation for AO/OTA 41-C2 tibial plateau fractures. We hypothesized that the plate–nail fixation would be superior to dual-plate fixation in this experiment.

## 2. Methods

### 2.1. Materials and Methods

**Figure 1:**
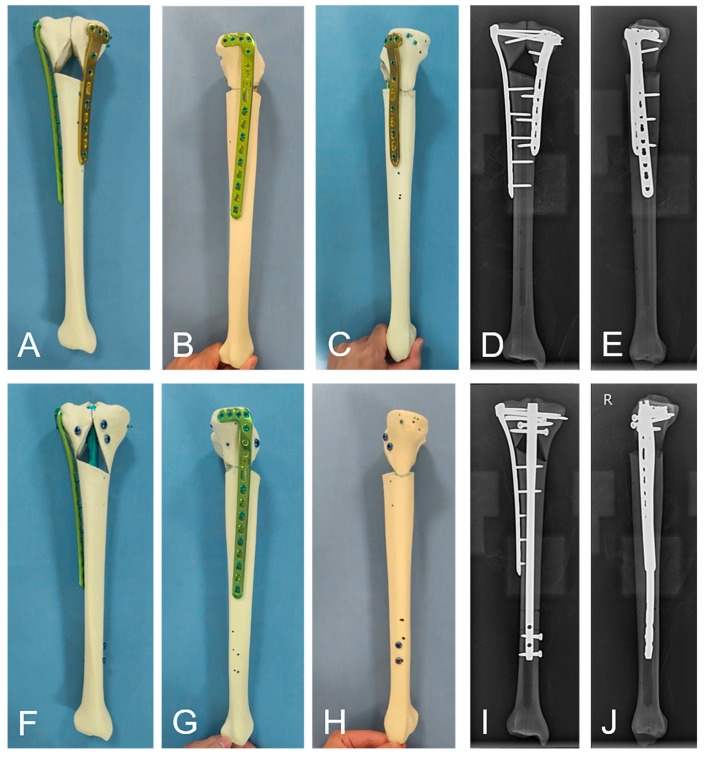
(**A**–**E**) Photographs and radiographic images depicting the fixation strategies of the dual-plate group. (**F**–**J**) Photographs and radiographic images depicting the fixation strategies of the plate–nail group.

Twenty artificial tibias (Type 1,110; SYNBONE AG, Malans, Switzerland; Length: 387 mm, Tibia plateau width: 74 mm, Shaft diameter: 27 mm, and Canal diameter: 8 mm) were selected for this study. From them, an AO/OTA 41-C2 tibial plateau fracture model was established according to the method described by Thamyongkit et al. [14]. The first fracture line was sawed vertically from the intercondylar eminence to simulate the articular surface fracture line of a C2 fracture. The second fracture line was sawed from the endpoint of the first fracture line at 20° from the tibial axis to the lateral cortex. The third fracture line was sawed inward from the endpoint of the first fracture line at 35° from the tibial axis. The fourth fracture line was sawed along the line connecting the fracture endpoints on the medial and lateral cortices, and the bone among the second, third, and fourth fracture lines was removed to simulate a comminuted AO/OTA 41-C2 tibial plateau fracture. Then, all the artificial tibia fracture models were randomly divided into two groups, which were fixed with the dual-plate and plate–nail methods, respectively. In the plate–nail group, the lateral plate (Double Medical, Xiamen, China; LSLP 13 Minimally Invasive Type I; 13 holes) was first used to maintain alignment at the anteroposterior and medial–lateral planes of the tibia. Three bicortical screws were used to fix the posterior screw hole at the proximal end of the plate, and then, three single-cortical screws were used to fix the distal end and maintain the position and alignment of the tibia. Then, a suprapatellar intramedullary nail (Double Medical, Xiamen, China; B-MIN-02 Tibial Locking Intramedullary Nail B; 9 × 280 mm) was inserted from the standard intramedullary nail entry point to maintain reduction. The distal end of the nail was fixed with two screws, and the proximal end was fixed with three screws. Finally, two bicortical screws were used to fix the middle part of the lateral plate, and two short screws were used to fix the proximal anterior screw hole because it was blocked by intramedullary nails. The dual-plate group model was reduced and fixed using the lateral plate (Double Medical, Xiamen, China; LSLP 13 Minimally Invasive Type I; 13 holes) first, and then the medial plate (Double Medical, Xiamen, China; LCLP 12 Minimally Invasive Type I; 8 holes) was added for maintenance reduction (Figure 1). Differently from the plate–nail group, the 10 screws of the lateral plate and the 8 screws of the medial plate were all bicortical. All model construction and surgical operations were completed by the same senior orthopedic surgeon according to standard procedures. After model construction and internal fixation installation, the distal bone of the model was fixed to the platform of a biomechanical testing machine using Denture Base Materials (Xinshiji, Type II, Shanghai, China). The results of the model under different stress conditions were recorded during the experiment.

### 2.2. Biomechanical Testing

**Figure 2:**
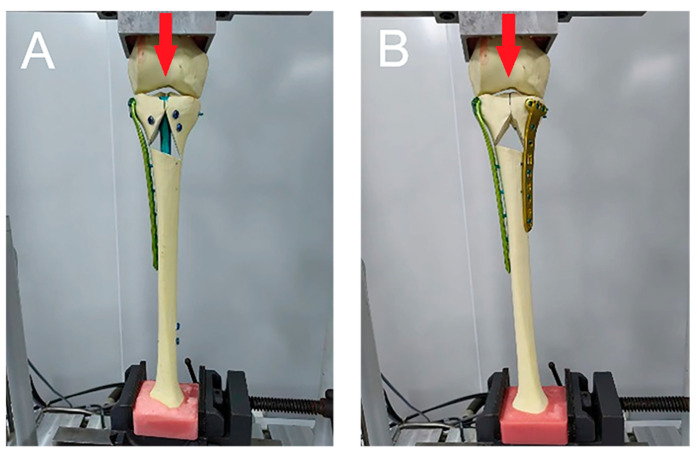
(**A**) A picture of a sample from the plate–nail group in the axial stress test. (**B**) A picture of a sample from the dual-plate group in the axial stress test. Red arrow: Force direction.

**Figure 3:**
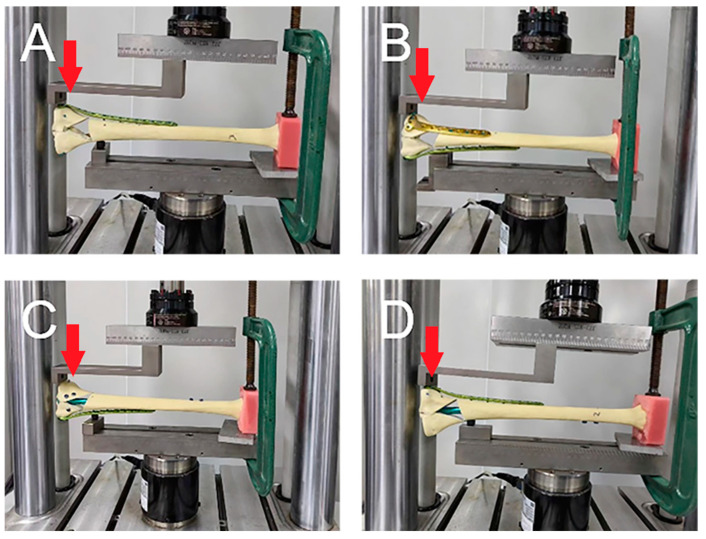
(**A**,**B**) Pictures of samples of the dual-plate group in the resisting varus and resisting valgus tests. (**C**,**D**) Pictures of samples of the plate–nail group in the resisting varus and resisting valgus tests. Red arrow: Force direction.

All experiments were performed at the Xiamen Medical Device Research and Development Testing Center using a mechanical testing machine (MTS Bionix Servo-hydraulics Test Systems Model 370.02; MTS Systems, Eden Prairie, MN, USA). Before the formal test, a 0–350 N axial compression load was applied to the tibia model for 30 s; this step was repeated three times, and time effects such as specimen relaxation and creep were eliminated by using this preloading approach. A uniform axial compression load of 0–800 N and a three-point bending load, respectively, were applied to each tibia. A 0–800 N axial compression load was gradually increased from 0 N to 800 N at a speed of 50 mm/min according to the knee joint load when standing on one limb, and data were recorded at 200 N, 400 N, 600 N, and 800 N (Figure 2) [18]. Due to the high varus deformity rate of single-plate fixation reported in the previous literature, we compared the resisting varus and resisting valgus abilities of the two groups to determine whether the plate–nail group had sufficient resisting bend ability. The 0–800 N bending load was tested twice, on the lateral and medial of the specimen, respectively, to simulate the specimens’ resisting bending stress under the two conditions of varus and valgus. The tibia is not only subjected to axial pressure but also requires a certain amount of intorsion and extorsion pressure during daily activities. Thus, in this study, the tibia was subjected to torsional loads at a rate of 2 °C/min, and the applied load was measured when the tibia twisted at 1°, 2°, 3°, 4°, and 5° (Figure 3). Finally, an axial load was applied to each tibia in each group, with increments of 10 N/s, until failure occurred, and the ultimate load was recorded. The results under the axial compressive load and torsional load were recorded, and the stiffnesses of the axial compression and torsion were calculated as the load (N) divided by the displacement (mm) (N/mm) and the load (N) divided by the degree (N/degree), respectively. In this study, the criteria for determining the stability failure of the fixation–bone–structure complex under stress were as follows: fracture, the loosening of the screws, or displacement of the proximal fracture fragment of greater than 5 mm.

### 2.3. Statistical Analysis

SPSS 24.0 software was used for statistical analysis of the data. The Shapiro–Wilk test was used to check the normality for continuous variables. Normally distributed continuous variables were described as mean ± standard deviation. One-way analysis of variance was performed to compare the differences in compression stiffness, compression displacement, ultimate compression load, valgus load, varus load, torsional stiffness, and torsional load. *p* < 0.05 was considered statistically significant.

## 3. Results

In this experiment, there were no loosened screws or fractures in any group of specimens. There was also no loosening between the specimens and the biomechanical testing machine, and no new fractures were found in the other parts of the specimens.

**Figure 4:**
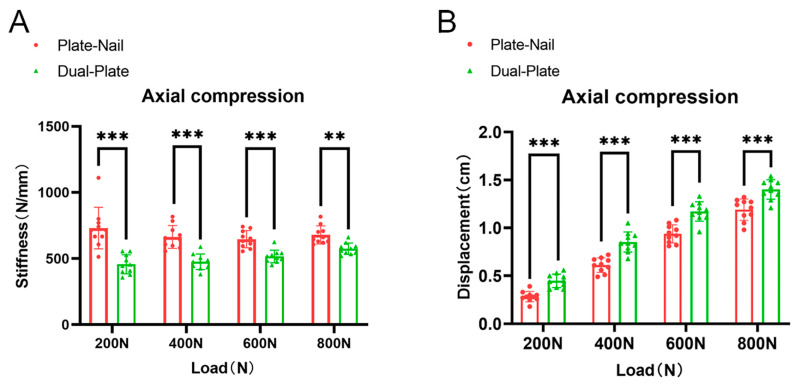
(**A**) The axial stiffness of the plate–nail group was significantly higher than that of the dual-plate group. (**B**) The displacement of the plate–nail group under axial load was significantly smaller than that of the dual-plate group. ** *p* < 0.01; *** *p* < 0.001.

In the axial compression test, the average stiffnesses of the plate–nail group under axial loads of 200 N, 400 N, 600 N, and 800 N were 730 ± 157 N/mm, 663 ± 87 N/mm, 646 ± 65 N/mm, and 678 ± 68 N/mm, respectively. The average stiffnesses of the dual-plate group were 475 ± 72 N/mm, 475 ± 59 N/mm, 516 ± 48 N/mm, and 574 ± 43 N/mm, respectively. The stiffnesses of the plate–nail group were significantly greater than those of the dual-plate group, and the results were statistically significant (*p* ˂ 0.05) (Figure 4A). The displacements of the plate–nail group under axial loads of 200 N, 400 N, 600 N, and 800 N were 0.28 ± 0.05 cm, 0.61 ± 0.07 cm, 0.94 ± 0.09 cm, and 1.19 ± 0.11 cm, respectively. The displacements of the dual-plate group were 0.45 ± 0.07 cm, 0.85 ± 0.09 cm, 1.17 ± 0.11 cm, and 1.41 ± 0.12 cm, respectively. The displacements of the plate–nail group were significantly smaller than those of the dual-plate group, and the results were statistically significant (*p* ˂ 0.05) (Figure 4B).

**Figure 5:**
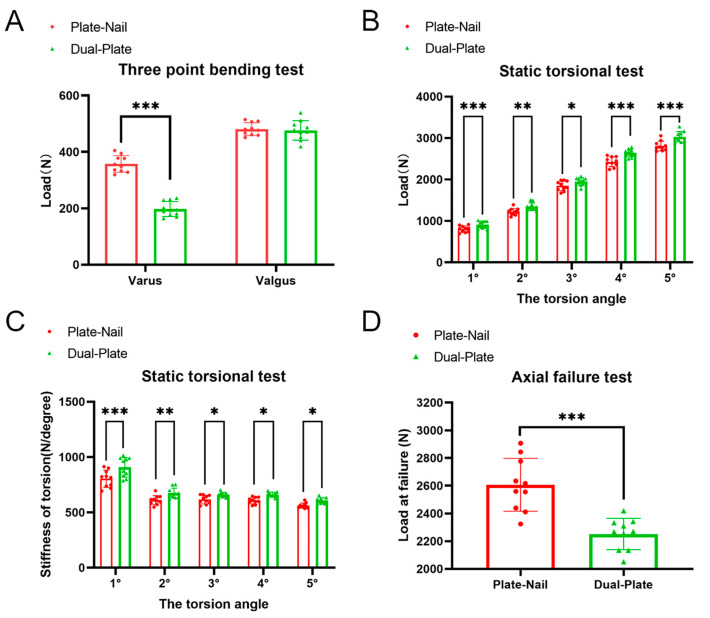
(**A**) In the resisting varus test, when the displacement of the proximal bone was >5 mm, the load of the plate–nail group was significantly greater than that of the dual-plate group. In the resisting valgus test, when the displacement of the proximal bone was >5 mm, the load of the plate–nail group was slightly greater than that of the dual-plate group. (**B**) In the torsion test, the load of the plate–nail group was significantly greater than that of the dual-plate group, and (**C**) the torsional stiffness of the plate–nail group was significantly greater than that of the dual-plate group. (**D**) The results of the axial failure test show that the ultimate load of the plate–nail group was significantly higher than that of the dual-plate group. * *p* < 0.05; ** *p* < 0.01; *** *p* < 0.001.

In the three-point bending test, when 800 N of valgus or varus stress was applied to the proximal tibial fracture fragments, stability failure (displacement of greater than 5 mm) occurred in either group before the bending stress reached 800 N. When valgus stress was used, the stress when the displacement of the specimens in the plate–nail group reached 5 mm was 357 ± 30 N, while in the dual-plate group, it was 197 ± 27 N. The plate–nail group had significantly greater resisting valgus stress than the dual-plate group, and the difference was statistically significant (*p <* 0.05). When varus stress was applied, the stress when the displacement of the specimens in the plate–nail group reached 5 mm was 481 ± 23 N, while in the dual-plate group, it was 477 ± 35 N. The plate–nail group had slightly stronger resisting varus stress than the dual-plate group, but the difference was not statistically significant (*p* > 0.05) (Figure 5A).

In the torsion test, the proximal tibia was rotated at a rate of 2°/min. At rotations of 1°, 2°, 3°, 4°, and 5°, the corresponding loads applied to the plate–nail group were 804 ± 77 N, 1221 ± 83 N, 1847 ± 117 N, 2428 ± 118 N, and 2808 ± 118 N, respectively. For the dual-plate group, the loads were 908 ± 88 N, 1348 ± 91 N, 1951 ± 90 N, 2636 ± 92 N, and 3031 ± 126 N, respectively. The dual-plate group had significantly greater loads than the plate–nail group, and the difference was statistically significant (*p <* 0.05) (Figure 5B). At rotations of 1°, 2°, 3°, 4°, and 5°, the corresponding stiffnesses applied to the plate–nail group were 804 ± 77 N/degree, 611 ± 42 N/degree, 616 ± 39 N/degree, 607 ± 29 N/degree, and 562 ± 23 N/degree, respectively. For the dual-plate group, the stiffnesses were 908 ± 88 N/degree, 674 ± 46 N/degree, 658 ± 22 N/degree, 659 ± 23 N/degree, and 606 ± 25 N/degree, respectively. The dual-plate group had significantly greater stiffnesses than the plate–nail group, and the difference was statistically significant (*p <* 0.05) (Figure 5C).

In the axial compression failure test, the average ultimate load (displacement of greater than 5 mm) of the specimens in the plate–nail group was 2607 ± 191 N, while in the dual-plate group, it was 2251 ± 113 N. The failure load of the plate–nail group was significantly higher than that of the dual-plate group, and the difference was statistically significant (*p <* 0.05) (Figure 5D).

## 4. Discussion

AO/OTA 41-C2 tibial plateau fractures are usually caused by high-energy trauma [1,2,3]. Many studies have reported that the infection rate of dual-plate fixation is significantly higher than that of single-plate fixation [11,19,20]. For this type of fracture, the metaphysis is seriously shattered and lacks bone support [21,22]. If fixation methods such as single plates and intramedullary nails are used, the fixation strength will be insufficient, which is likely to lead to postoperative reduction failure for which dual-plate fixation is usually required [12,13]. However, high-energy fractures are often associated with severe soft tissue injuries such as subcutaneous ecchymosis, tension blisters, and open fractures [23,24,25]. If dual-plate fixation is used early after injury, the severely injured soft tissue conditions will be poor, making it difficult to place two plates, while the use of single-plate fixation or intramedullary nail fixation will lead to insufficient fixation strength and cause loss of postoperative reduction [12,13]. If early external fixation combined with limited internal fixation is used, or if surgery is used after the improvement of soft tissue conditions, the treatment time will be longer and could easily lead to complications associated with being bedridden, such as knee joint stiffness and muscle atrophy [26,27,28]. A recent study has reported that the use of intramedullary nail fixation was combined with plate fixation for tibial plateau fractures with reducible articular fracturing in 16 patients [15]. The fractures were healed in all patients, and only one patient had an incision infection, demonstrating good clinical efficacy. Although the current evidence recommends the use of dual-plate fixation for C2 and C3 tibial plateau fractures, this surgical technique is a better option for tibial plateau fractures with poor soft tissue conditions.

For C2 and C3 tibial plateau fractures, using only the lateral plate or intramedullary nails often leads to postoperative reduction loss due to insufficient fixation strength, resulting in varus deformity. Many works in the literature have demonstrated that adding a supporting plate on the medial side can effectively prevent postoperative reduction loss, but it is difficult to place dual plates in cases with poor soft tissue conditions [29,30,31,32]. The technique of the intramedullary nail combined with plate fixation, which places the intramedullary nail through the patellar approach to maintain the tibial axis instead of using a medial plate, can also avoid the medial soft tissue [15]. Biomechanically, the intramedullary nail has the strongest resisting axial displacement, while the plate has the strongest resisting torsion. However, although this technique has achieved good clinical efficacy in controlling tibial varus deformity by using the intramedullary nail instead of the medial plate when the medial plate cannot be placed, there is still a lack of biomechanical evidence to determine whether its fixation strength can replace the medial plate [17,33,34]. Therefore, we created a 41-C2 fracture model of artificial tibias to evaluate whether the intramedullary nail combined with plate fixation can achieve the same biomechanical strength as dual-plate fixation.

In our experimental results, we found that there was no significant statistical difference in the displacement results between the two groups in the resisting valgus test. The results indicated that the ability to control valgus deformity was similar in both groups with regard to the lateral plate and intramedullary nail fixation. In the resisting varus test, the group with the intramedullary nail combined with the plate showed significantly stronger resisting varus ability compared to the group with dual-plate fixation. This suggests that an intramedullary nail can effectively replace the medial buttress plate in controlling the tibial axis. This is inconsistent with previous reports stating that plates have stronger resisting varus ability than intramedullary nails. We believe that this is because the medial buttress plate that we used was relatively short and longer plates or nails can be used to increase the resisting varus strength. The tibia is not only subjected to axial pressure but also requires a certain amount of intorsion and extorsion pressure during daily activities. For this reason, we also conducted a torsional test, and the results showed that the dual-plate group had significantly stronger torsional resistance ability than the plate–nail group, which is consistent with the currently reported biomechanical results. The eccentric fixation of plates has a stronger rotational resistance ability than that of intramedullary nails located at the rotation center. Although most studies have shown that intramedullary nails have stronger axial compression resistance ability than plates, others have found that in distal femoral fracture models, dual-steel-plate fixation had stronger resisting axial displacement than intramedullary nails combined with steel plates. In the results of our axial compression and failure experiment, the plate–nail group had observably significantly stronger axial compression resistance ability and stiffness than the dual-plate group. The biomechanical results showed that the combination of plate and intramedullary nail fixation had higher stability than dual-plate fixation in the treatment of 41-C2 tibial plateau fractures.

This study had some limitations. As the soft tissue surrounding the knee and the role of the fibula were not considered, the artificial bone model could not fully simulate the situation in vivo. Compared to in vitro cadaver studies, although artificial bone has better consistency of bone quality, physiological changes in human bone density and force distribution were not considered, and fatigue tests could not be performed on this artificial bone model [35]. This study has found that in addition to resisting torsion ability, the intramedullary nailing combined plate is no lesser or better than dual-plate fixation in terms of resisting axial and resisting bend stress, but the excessive stiffness is not a disadvantage for secondary fracture healing [36,37]. For patients with normal bone mass and osteoporosis, whether this operation can be put onto the ground early and whether it can achieve better fracture healing still need further research [18,38,39]. Although the intramedullary nailing technique has higher axial stiffness and causes less soft tissue damage, it has poor fixation ability on the proximal fracture block, especially when the articular surface is crushed. The proximal locking screw of the intramedullary nailing may not exert a good holding effect on the crushed fracture block, which will seriously affect the fixation effect of the nailing. Therefore, although this procedure is suitable for C2 and some C3 fractures whose articular surfaces can be reduced, this study did not take into account those C3 fractures, and more trials are needed to perform biomechanical tests that will prove this conclusion.

## 5. Conclusions

The experimental results revealed that during both the axial compression and the axial compression failure tests, the plate–nail group exhibited higher stiffness and smaller displacement relative to the dual-plate group, suggesting superior axial stability in the plate–nail group. Contrarily, the results of the resisting torsion test indicated that the dual-plate group had a significantly greater capacity to resist torsion compared to the plate–nail group. Nonetheless, in the resisting varus test, the plate–nail group demonstrated enhanced stability, implying a more effective resistance to tibial varus deformation when compared to the dual-plate group. Additionally, both groups showed comparable levels of resistance to valgus deformation.

## Data Availability

The data presented in this study are available from the corresponding author.
